# Supplementary material for: The Effect of Pre-Operative Verbal Confirmation for Interventional Radiology Physicians on Their Use of Personal Dosimeters and Personal Protective Equipment
Source: Int J Environ Res Public Health. 2022 Dec 15;19(24):16825. doi: 10.3390/ijerph192416825 (PMC9778706; doi:10.3390/ijerph192416825)
Supplement: Supplementary file 1 [file ijerph-19-16825-s001.zip › ijerph-1923411-supplementary.pdf]

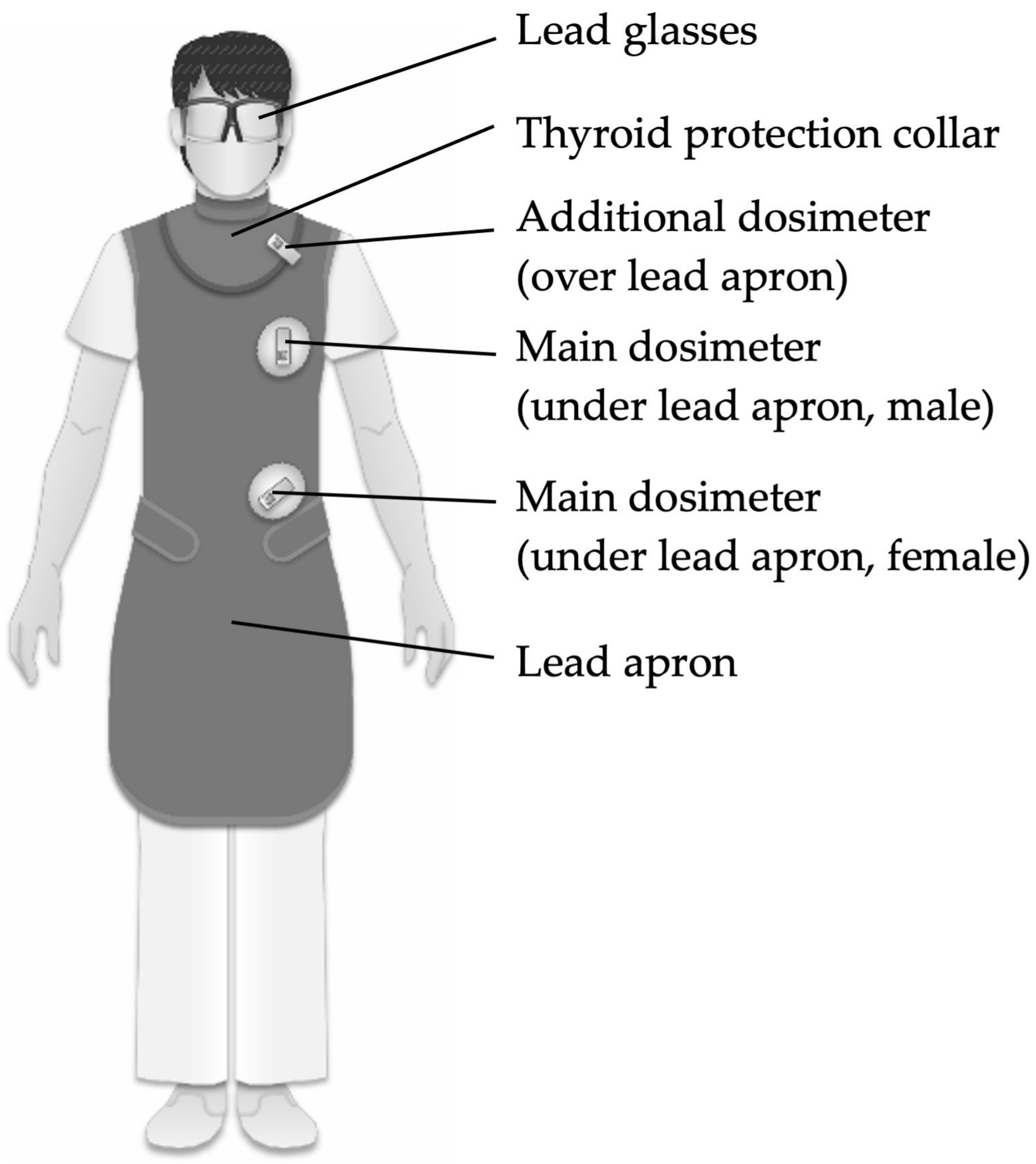

Figure S1. The personal passive dosimeter and the personal protective equipment the physicians surveyed wore in this analysis. Due to the high radiation doses of physicians, it is recommended to wear a lead apron and thyroid protection collar to reduce the risk of cancer (stochastic effect). Moreover, wearing lead glasses is recommended to avoid cataracts (tissue reaction). Japanese law stipulates the installation of the main dosimeter and the additional dosimeter as needed. The main dosimeter is to be worn inside the lead apron (male on the chest, female on the abdomen), and the additional dosimeter is to be worn outside the lead apron.

Medical treatment date and time<br>(Year) (Month) (Day) (Start time)	Patient information<br>Name : (M ・ F) Age : years old	Inspector<br>Name:
Procedure name :	(inspection or treatment)	

No	Physician name	Main dosimeter (under lead apron)	Additional dosimeter (over lead apron)	Lead apron	Lead glasses	Thyroid protection collar	Remarks
1	aaaaaaa	Y or N	Y or N	Y or N	Y or N	Y or N	
2	bbbbbbb	Y or N	Y or N	Y or N	Y or N	Y or N	
3	ccccccc	Y or N	Y or N	Y or N	Y or N	Y or N	
4	ddddddd	Y or N	Y or N	Y or N	Y or N	Y or N	
5	eeeeeee	Y or N	Y or N	Y or N	Y or N	Y or N	

Figure S2. The radiation work checklist. The inspector, a radiological technologist overseeing the process, conducted the visual inspection during the pre-intervention and intervention period. Physicians' wearing status of the personal dosimeter (main and additional) and personal protective equipment (lead apron, lead glasses, and thyroid protection collar) were inspected for each procedure. Patient characteristics and the procedure name with its purpose, for inspection only or treatment, were also included in the checklist.

Table S1. Use rate of personal passive dosimeters by physicians

Physician (n = 14)	Clinical department	Number of visual inspection			Main dosimeter (under lead apron)					Additional dosimeter (over lead apron)				
					Pre-intervention period		Intervention period			Pre-intervention period		Intervention period		
		Pre-intervention period	Intervention period	*p* value†	Number of times used	Use rate (%)	Number of times used	Use rate (%)	*p* value†	Number of times used	Use rate (%)	Number of times used	Use rate (%)	*p* value†
a, M	Neurosurgery	14	10		9	64	9	90		9	64	9	90	
b, M	Neurosurgery	36	29		27	75	29	100		27	75	29	100	
c, M	Neurosurgery	41	4		18	44	4	100		18	44	4	100	
d, M	Internal Medicine	32	39		19	59	39	100		19	59	39	100	
e, M	Cardiology	35	18		25	71	18	100		25	71	18	100	
f, M	Internal Medicine	60	38		60	100	38	100		60	100	38	100	
g, M	Endocrinology	3	3		0	0	2	67		0*	0*	2**	67**	
h, M	Orthopedic surgery	16	3		1	6	3	100		0*	0*	3**	100**	
i, M	Orthopedic surgery	92	75		15	16	73	97		0*	0*	59**	79**	
j, M	Orthopedic surgery	58	72		46	79	72	100		0*	0*	53**	74**	
k, M	General Surgery	9	22		1	11	22	100		0*	0*	0*	0*	
l, M	General Surgery	14	19		2	14	19	100		0*	0*	0*	0*	
m, M	Urology	9	18		5	56	18	100		0*	0*	0*	0*	
n, F	Urology	5	15		3	60	15	100		0*	0*	0*	0*	
Mean ± SD		30.3 ± 25.8	26.1 ± 23.1	0.382	16.5 ± 18.2	47 ± 32	25.8 ± 22.9	97 ± 9	0.002	11.3 ± 17.4	30 ± 37	18.1 ± 21.3	65 ± 44	0.008
Median [range]		24 [3–92]	18.5 [3–75]		12 [0–60]	57 [0–100]	18.5 [2–73]	100 [67–100]		0 [0–60]	0 [0–100]	6.5 [0–59]	85 [0–100]	

SD: standard deviation

M: Male

F: Female

* Under the operation with single-dosimeter approach

** Switched management method from single-dosimeter to double-dosimeter approach during intervention period

† Wilcoxon's signed rank test.

Table S2. The use rate of personal protective equipment (PPE) by physicians

Physician (n = 14)	Clinical department	Number of visual inspections			Lead apron					Lead glasses					Thyroid protection collar				
					Pre-intervention period		Intervention period			Pre-intervention period		Intervention period			Pre-intervention period		Intervention period		
		Pre-intervention period	Intervention period	*p* value†	Number of times used	Use rate (%)	Number of times used	Use rate (%)	*p* value†	Number of times used	Use rate (%)	Number of times used	Use rate (%)	*p* value†	Number of times used	Use rate (%)	Number of times used	Use rate (%)	*p* value†
a, M	Neurosurgery	14	10		14	100	10	100		13	93	10	100		13	93	9	90	
b, M	Neurosurgery	36	29		36	100	29	100		33	92	29	100		35	97	28	97	
c, M	Neurosurgery	41	4		41	100	4	100		30	73	4	100		39	95	4	100	
d, M	Internal Medicine	32	39		32	100	39	100		9	28	39	100		32	100	39	100	
e, M	Cardiology	35	18		35	100	18	100		3	9	18	100		29	83	16	89	
f, M	Internal Medicine	60	38		60	100	38	100		58	97	38	100		60	100	38	100	
g, M	Endocrinology	3	3		3	100	3	100		3	100	2	67		3	100	3	100	
h, M	Orthopedic surgery	16	3		16	100	3	100		0	0	3	100		0	0	3	100	
i, M	Orthopedic surgery	92	75		92	100	75	100		0	0	75	100		0	0	75	100	
j, M	Orthopedic surgery	58	72		58	100	72	100		13	22	72	100		2	3	72	100	
k, M	General Surgery	9	22		9	100	22	100		0	0	21	95		0	0	21	96	
l, M	General Surgery	14	19		14	100	19	100		0	0	17	89		0	0	17	90	
m, M	Urology	9	18		9	100	18	100		0	0	18	100		5	56	18	100	
n, F	Urology	5	15		5	100	15	100		0	0	15	100		0	0	15	100	
Mean ± SD		30.3 ± 25.8	26.1 ± 23.1	0.382	30.3 ± 25.8	100 ± 0	26.1 ± 23.1	100 ± 0	1.000	11.6 ± 17.4	37 ± 43	25.8 ± 23.3	97 ± 9	0.003	15.6 ± 19.7	52 ± 48	25.6 ± 23.3	97 ± 4	0.008
Median [range]		24 [3–92]	18.5 [3–75]		24 [3–92]	100	18.5 [3–75]	100		3 [0–58]	15 [0–100]	18 [2–75]	100 [67–100]		4 [0–60]	69 [0–100]	17.5 [3–75]	100 [89–100]	

SD: standard deviation

M: Male

F: Female

† Wilcoxon's signed rank test.

Table S3. Personal exposure dose and dose rate of physicians

Physician (n = 14)	Clinical department	Number of radiation medical treatments conducted			Total fluoroscopy time (min) (a)			Effective dose (mSv) (b)			Effective dose rate (μSv/min) (b/a)			Lens equivalent dose (mSv) (c)			Lens equivalent dose rate (μSv/min) (c/a)		
		Pre-intervention period	Intervention period	*p* value†	Pre-intervention period	Intervention period	*p* value††	Pre-intervention period	Intervention period	*p* value†	Pre-intervention period	Intervention period	*p* value†	Pre-intervention period	Intervention period	*p* value†	Pre-intervention period	Intervention period	*p* value†
a, M	Neurosurgery	22	15		259.8	238.0		0.3	0.4		1.2	1.7		0.7	0.9		2.7	3.8	
b, M	Neurosurgery	56	36		1027.7	957.6		1.4	1.3		1.4	1.4		7.5	7.8		7.3	8.1	
c, M	Neurosurgery	57	5		1041.7	116.1		0.4	0.1		0.4	0.9		1.0	0.8		1.0	6.9	
d, M	Internal Medicine	57	48		946.8	643.4		1.1	4.0		1.2	6.2		2.0	7.1		2.1	11.0	
e, M	Cardiology	52	23		1926.7	647.3		3.2	1.0		1.7	1.5		7.3	3.6		3.8	5.6	
f, M	Internal Medicine	108	67		2901.5	934.1		6.3	4.8		2.2	5.1		23.7	13.9		8.2	14.9	
g, M	Endocrinology	4	6		231.5	274.6		0.4	0.5		1.7	1.8		0.4	1.7		1.7	6.2	
h, M	Orthopedic surgery	19	3		18.0	2.8		0.0	0.0		0.0	0.0		0.0	0.1		0.0	35.7	
i, M	Orthopedic surgery	118	77		N/A	97.6		0.0	3.2		0.0	32.8		0.0	12.0		0.0	123.0	
j, M	Orthopedic surgery	100	53		88.2	45.5		1.2	1.5		13.6	33.0		1.4	7.6		15.9	167.0	
k, M	General Surgery	23	44		267.3	348.1		0.1	1.5		0.4	4.3		0.1	1.6		0.4	4.6	
l, M	General Surgery	35	30		307.5	146.7		0.2	1.1		0.7	7.5		0.2	1.2		0.7	8.2	
m, M	Urology	18	21		135.6	129.1		0.6	0.4		4.4	3.1		0.6	0.4		4.4	3.1	
n, F	Urology	8	18		30.5	129.2		0.1	0.4		3.3	3.1		0.1	0.4		3.3	3.1	
Mean ± SD		48.4 ± 37.4	31.9 ± 23.3	0.030	706.4 ± 865.9	336.4 ± 324.8	0.344	1.1 ± 1.7	1.4 ± 1.5	0.345	2.3 ± 3.5	7.3 ± 11.0	0.033	3.2 ± 6.4	4.2 ± 4.6	0.124	3.7 ± 4.3	28.7 ± 50.7	0.003
Median [range]		43.5 [4–118]	26.5 [3–77]		267.3 [18.0–2901.5]	192.4 [2.8–957.6]		0.4 [0.0–6.3]	1.1 [0.0–4.8]		1.3 [0.0–13.6]	3.1 [0.0–33.0]		0.7 [0.0–23.7]	1.7 [0.1–13.9]		2.4 [0.0–15.9]	7.5 [3.1–167.0]	

SD: standard deviation
M: Male
F: Female
N/A: not aveilable
† Wilcoxon's signed rank test
†† Mann-Whitney's U test.
